# The Role of Wideband Tympanometry in the Diagnosis of Meniere's Disease

**DOI:** 10.3389/fneur.2022.808921

**Published:** 2022-01-27

**Authors:** Xiangming Meng, Kangxu Zhu, Jing Yue, Chengzhou Han

**Affiliations:** Department of Otolaryngology, Wuxi Huishan District People's Hospital, Wuxi, China

**Keywords:** Meniere's disease, wideband tympanometry, endolymphatic hydrops, inner ear, absorbance, eustachian tube dysfunction

## Abstract

Meniere's disease (MD) is a clinical syndrome characterized by spontaneous recurrent vertigo, usually accompanied by hearing loss, tinnitus, and aural fullness. The cause of MD remains unclear and is generally considered to be associated with endolymphatic hydrops. Studies showed that patients with MD could have eustachian tube dysfunction (ETD). ETD can disrupt the pressure balance between the middle and inner ear and impair the inner ear's function. In recent years, several studies have attempted to identify MD by using wideband tympanometry (WBT). However, there are limited studies in this area. There is no consensus on how to use WBT to diagnose Meniere's disease. Therefore, we endeavored to conduct a narrative review in this aspect based on the latest research findings. Reduction in resonance frequency and absorbance are characteristic of MD and can identify Meniere's disease. The use of an increase in the integrated area of absorbance as an indicator for identifying MD is controversial. WBT seems to be ineffective as a diagnostic tool during the acute episodes of Meniere's disease. Patients with MD may benefit from WBT. WBT has excellent potential for future use in Meniere's disease. However, further large sample sizes, multicenter studies are needed.

## Introduction

Meniere's disease (MD) is a clinical syndrome characterized by episodes of recurrent spontaneous vertigo, usually accompanied by fluctuating sensorineural hearing loss, tinnitus, and aural fullness (1). This syndrome was initially described by a French doctor named Prosper Meniere in 1861 (2). The prevalence of MD varies widely worldwide, with estimates ranging from 3.5 per 100,000 to 513 per 100,000 individuals (3). MD seriously affects patients' quality of life and produces substantial direct or indirect health care costs (3). The cause of MD remains unknown, but it is considered a multifactorial disease caused by the interaction of genetic, anatomical, autoimmune, and environmental factors (4). Studies have proved that MD is associated with endolymphatic hydrops (EH), which cause an enlarged endolymphatic space in the inner ear (IE) (1, 5). The presence of EH can be verified non-invasively by intravenous injection of delayed gadolinium-enhanced magnetic resonance imaging of the IE (6). The measurement of intralabyrinthine pressure allows a better understanding of MD (7). Currently, techniques such as vestibular evoked myogenic potentials (VEMP), electrocochleography (ECoG), distortion product otoacoustic emissions (DPOAE), and caloric test have been used to assess the condition of MD (8, 9). A study investigating 18F-FDG cerebral uptake in patients with MD vs. healthy controls found lower cortical activity in areas such as the Heschl's gyrus, the posterior part of the insula, and thalamus in patients with MD compared to normal controls (10). A recent study revealed a significant correlation between low-, mid-, and high-tone hearing thresholds and the grading of hydrops in the cochlea and vestibule (11). Consequently, fluctuating hearing loss in patients with MD may be associated with EH as well as other factors.

In recent years, the possibility of multifrequency tympanometry (MFT) as a new diagnostic tool for MD has been evaluated. The use of MFT to investigate experimentally induced cricoid ligament alterations in guinea pigs revealed significant 2 kHz tympanic conduction curves, which could imply IE pressure (12). Although some meaningful results have been obtained, diagnostic accuracy remains limited (13). The concept of wideband tympanometry (WBT) was first proposed in the 1980s (14). Keefe et al. developed a test method for WBT and obtained data for clinical measurements (15). WBT can provide more sensitive and specific results for middle ear (ME) pathologies such as secretory otitis media, otosclerosis, and ossicular chain disruption, thus helping clinicians differentiate diagnoses (16–18). Changes in IE compartment pressure can induce symptoms in some IE diseases, and WBT has been found to be helpful in the pathological diagnosis of elevated intracranial pressure and IE pressure (8). MD can also cause changes in ME conduction (19). Therefore, several researchers have recently attempted to identify MD by using WBT. However, research is scarce in this field, which is still poorly understood. There is no agreement on how WBT should be used to diagnose MD.

To further the understanding of WBT for diagnosing MD, we have endeavored to produce a narrative review based on the most recent research findings. We searched a variety of literature databases, including PubMed, Scopus, Web of Science, Embase, and Cochrane, using variations of the descriptors WBT, MD, and EH. The inclusion criteria were literature on the use of WBT for identifying MD, which was published in English or Chinese. Non-relevant topic studies, non-English or non-Chinese language articles were excluded. Ultimately, 7 studies met the criteria for inclusion. In this mini-review, we will highlight the role of WBT in the diagnosis of MD.

## Subsections Relevant for the Subject

### Properties and Advantages of Wideband Tympanometry

Traditional tympanometry assesses the impedance of the ME at a frequency of 226 Hz. Still, this traditional measurement method yields different results depending on the anatomical characteristics of the ME cavity, which may influence the test results (20). WBT employs 1/24-octave frequency intervals ranging from 226 to 8,000 Hz, delivered into the ear canal by a descending pressure sweep between + 250 to −350 dPa (4). Therefore, WBT is less vulnerable to myogenic noise from the patient movement since the transient stimulus involves multiple frequencies (20). As a result, WBT provides a more trustworthy diagnostic value than traditional tympanometry.

**Figure 1:**
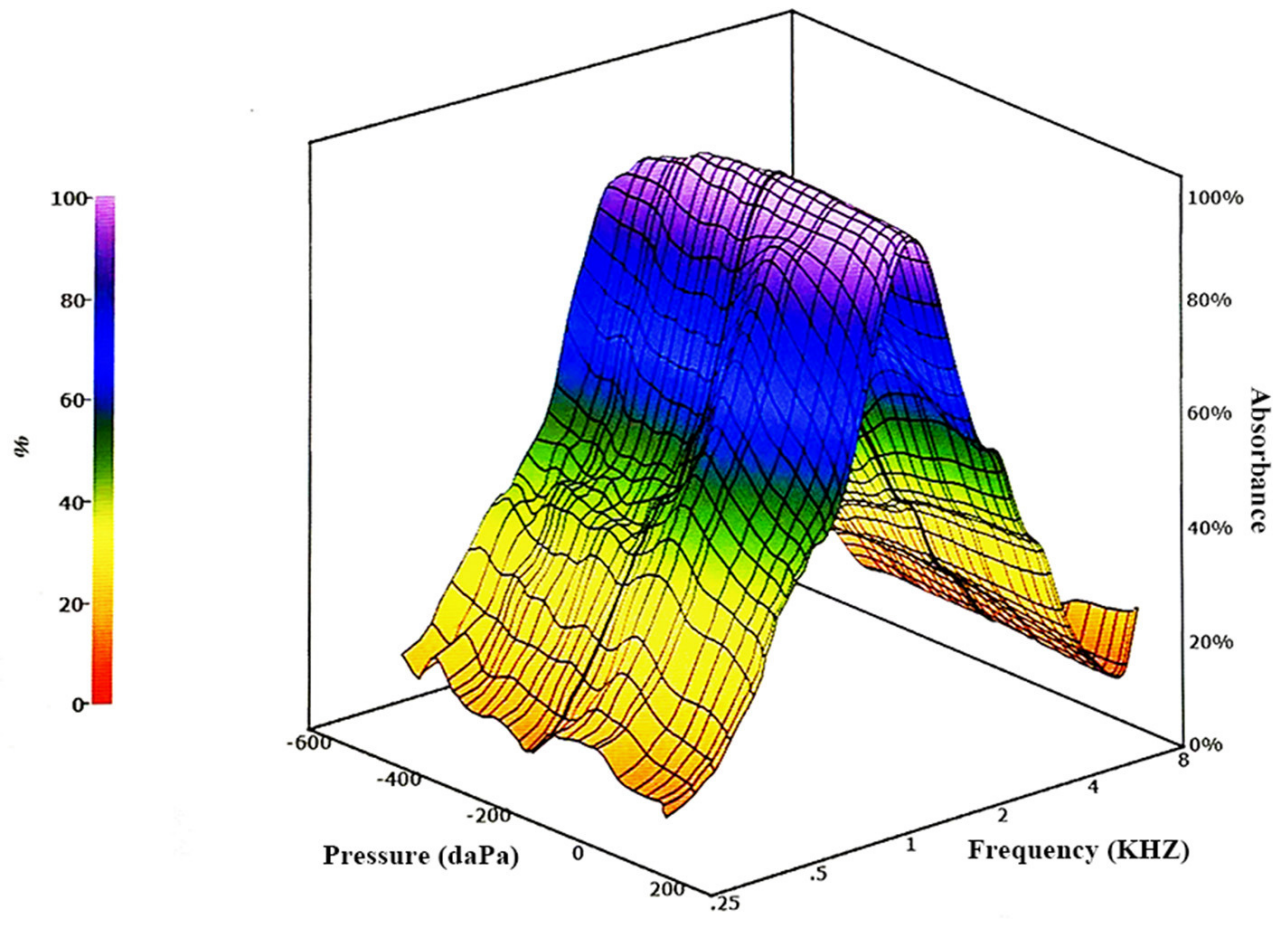
Figure showing a three-dimensional broadband tympanometry image with simultaneous absorbance, frequency, and pressure data.

In a WBT test, a microphone-generated sound signal is sent through the external ear canal to the ME, where a portion of it is absorbed, referred to as absorbed sound energy; the remaining portion is reflected by the tympanic membrane, which is referred to as reflected sound energy. The microphone can collect reflected sound energy in the external ear canal, and the absorbed sound energy can be exhibited indirectly via reflected sound energy. The ratio of absorbed sound energy to total sound energy is known as absorbance. The following formula calculates the absorbance. Absorbance (energy) = absorbed power/incident power = 1 – energy reflectance (4). The detection of sounds at different frequencies of WBT can be affected by various ME pathologies, and EA values can reflect abnormalities in the external ear canal and ME (14). Results of WBT measurements are displayed in a three-dimensional figure with simultaneous display of data such as absorbance, frequency, and pressure (Figure 1). Various forms of information, such as absorbance, tympanometric peak pressure, and traditional tympanometry, are also available in a single recording. Therefore, WBT addresses a number of the drawbacks and limitations associated with traditional tympanometry.

### Relationship Between Meniere's Disease and Eustachian Tube Dysfunction

There may be a link between MD and eustachian tube dysfunction (ETD). The eustachian tube is a natural pressure regulating system that balances the pressure in the ME to the external pressure. It plays a vital role in the ventilation and drainage of the tympanic cavity and the mastoid air cell system. ETD can disrupt the pressure balance between the ME and the IE, impairing the function of the IE (21, 22). The partial pressure of oxygen in the ME is low, significantly lower than the atmospheric partial pressure (23). The oxygen used in the IE is derived in part from the oxygen supply from the ME. Hypoxia may exist in the hydropic IE (23). In a hypoxic state, the pressure of the lymphatic fluid in the IE increases, further aggravating the existing EH and worsening the condition (24). Thus, inserting a ventilation tube (VT) can increase the oxygen supply to the ME and IE, thereby alleviating MD symptoms.

Patients with MD may be associated with ETD. According to a study by Kitajima et al., ~25% of patients with MD exhibit ETD (21). However, this opinion is highly controversial. It is uncommon to observe adult MD patients with secretory otitis media related to ETD; therefore, the 25% prevalence rate may be exaggerated. Brattmo et al. discovered a significant resistance to opening the eustachian tube in three out of four different provocation tests in their investigation of ME pressure in subjects with MD (19). Park et al. used direct sonotubometric measures to assess the eustachian tube function of MD patients and discovered that these patients showed mild ETD (25). Additionally, the severity of MD is associated with the occurrence of ETD; the more severe the stage, the higher the incidence (21).

VT can affect the pressure mechanism in the IE through the round window membrane. Several studies have demonstrated that inserting a VT is beneficial in treating some patients who have MD. According to a prospective, randomized study, patients with MD who underwent endolymphatic sac shunts and patients who received VT inserted into the tympanic membrane had significantly fewer vertigo episodes at 6 and 12 months post-operatively with no difference between the two treatments (22). The authors believed that in individuals with MD who have severe vertigo symptoms, a VT put into the tympanic membrane should be the primary treatment choice. If this treatment is ineffective, endolymphatic sac surgery should be considered (22). Sugawara et al. studied the long-term outcomes of seven patients with MD who were treated with the insertion of a VT into the tympanic membrane, finding that four patients had significant symptom control and three patients had limited symptom control following 42 months (24). Although this treatment has limited long-term success, it remains a viable therapeutic option for MD because of its simplicity and minimum invasiveness.

### Wideband Tympanometry for Meniere's Disease

WBT can be applied to the diagnosis of MD based on the assumption that the stapes footplate and the annular ligament serve as the loudspeaker of the IE (26). EH causes increased perilymphatic pressure, which pushes the stapes footplate toward the ME, limiting the movement of the ossicular chain and thus decreasing the compliance of the ME (26). Here, we highlight the performance and the significance of various WBT indicators and parameters in diagnosing MD.

#### Resonance Frequency

The resonance frequency (RF) of the affected side of MD patients was significantly lower than that of the asymptomatic ear of the unaffected side of MD patients and the control group (27). The cause of the affected ear's markedly decreased RF in MD patients may be associated with aberrant IE pressure (13). However, some studies suggest that the sensitivity of these data is insufficient for diagnosis and that WBT might be used as a supplementary assessment (13).

#### Absorbance

Recently, Miehe et al. found that patients with MD had significantly lower absorbance obtained by broadband tympanometry in the frequency range of 2,000–4,000 Hz compared to normal subjects (4). Since this study was a retrospective case-control study, its diagnostic criteria also changed over time, but there were no differences in mean absorbance measurements between all MD patients and the subgroup of patients who met the new criteria for MD. A cross-sectional study by Tanno et al. found significant differences in absorbance in the low-frequency region between symptomatic and asymptomatic patients with MD compared to normal individuals (28). WBT can be used to complement the diagnostic criteria for MD, which meets both the criteria of the previous and the new criteria for MD (4). In addition, WBT allows differentiating between asymptomatic and symptomatic MD patients (28).

#### Integral Area of the Absorbance

When applying WBT detection, 107 absorbance values from 226 to 8,000 Hz can be obtained, and then the integral area of the absorbance (IAA) of the subject is calculated. Two studies from China compared the difference in IAA between the affected ear and the unaffected ear in MD patients, yielding different results (27, 29). According to a study by Li et al., IAA in the symptomatic ear is larger than that in the asymptomatic ear in patients with MD, and the difference is statistically significant (29). However, Lan et al. found that IAA in the affected ear of patients with MD was not significantly different from that of the asymptomatic ear but only showed a slightly larger IAA in the affected ear than in the unaffected ear (27). Because of EH in the IE of patients with MD, there is more fluid in the membranous labyrinth on the affected side than on the healthy side. Acoustic energy is more readily absorbed as it passes through the liquid, resulting in a larger IAA in the affected ear than in the healthy ear (29). Whether the increase in IAA can be used to identify MD is controversial and requires further study.

#### G-Width

G-width is defined as the bimodal width of the waveform obtained when the conductance is measured at 2,000 Hz (30). Several earlier investigations employing MFT discovered a widened G-width in individuals with MD, which may be a diagnostic characteristic (30, 31). However, all the above studies were performed during the quiescence phase of MD. A recent prospective case-control study by Cakir Cetin et al. found no difference in G-width between the acute attacks phase patients with MD and healthy controls (26). Therefore, according to this study, WBT seems to have no diagnostic value in acute episodes of MD.

## Limitations of the Current Study and Prospects

WBT is a more comprehensive and relatively new indicator that may provide valuable information for patients than traditional tympanometry or MFT. It has the advantages of being objective, practical, minimally invasive, and rapid (28). However, WBT has been used mainly for research purposes, and its clinical application is still not widespread (4).

Currently, WBT has shown significant advantages in the diagnosis of ME diseases. The absorbance in the WBT systematically decreases with increasing ME effusion accumulation, which is a powerful and sensitive indicator of the volume of ME effusion in children with secretory otitis media (16). However, there are few studies using WBT to diagnose MD, and the sample sizes included in these studies are generally small. Most studies have only measured WBT in patients with MD at rest or the acute attacks phase of the condition, and follow-up WBT data of patients are lacking (13). There were some differences in the normative values of WBT in different age populations (32). In addition, the normative values of WBT parameters vary among different ethnic groups (33). However, no studies have employed WBT to compare ethnic groups of patients with MD. The establishment of a WBT dataset for a specific ethnically healthy adult population will help apply WBT in clinical practice while also serving as a reference for future studies (34).

Other tests have some advantages in diagnosing MD. The VEMP test is used to evaluate the saccule and utricle bilaterally by monitoring the sternocleidomastoid and inferior oblique muscles (4). The interaural amplitude difference ratio of VEMP correlates with the staging of MD and can be used as an auxiliary indicator to determine the stage of MD (35). On ECoG, a ratio of total sum potential to compound action potential >0.40 is regarded as a significant indicator of EH (8). Tone burst ECoG is more sensitive than gadolinium-enhanced MRI scans in diagnosing MD (36). In the DPOAE examination, the reduction of the signal-to-noise ratio at 1 kHz is of some value in the diagnosis of EH (8). Additionally, DPOAE detects cochlear ischemia in seconds and monitors cochlear blood flow indirectly for hearing protection during cerebellopontine angle surgery (37). The caloric test examines the vestibular function at low frequencies (4). According to a recent study, the caloric test complements the horizontal video head pulse test in assessing vestibular disease and plays an important role in suspected EH (9).

Although WBT has some value in the diagnosis of MD, it is essential to note that WBT measurements should be used as part of a test battery and interpreted in conjunction with the patient's history, physical examination, and other objective tests rather than relying solely on WBT results.

The wideband acoustic immittance database (WAI), funded by the National Institutes of Health, has facilitated the development of research in this area, intending to allow audiological researchers to share WAI measurement data and perform comprehensive over multiple datasets (38). Otolaryngologists should strengthen international cooperation, and future prospective multicenter studies on this issue, including larger sample sizes, should be conducted. At present, WBT has already shown a role in recognizing MD as an accurate and rapid test method. With more profound research and better understanding, WBT will have great potential for MD in the future and has a broad application prospect.

## Conclusions

In this review, we would like to emphasize the following points:

- Patients with MD may benefit from WBT, a more comprehensive and relatively new indicator that provides helpful information.
- Patients with MD are likely to have ETD, theoretically allowing for identifying the condition using WBT.
- The reduced resonance frequency and absorbance are characteristic of MD and can identify MD.
- WBT can detect between asymptomatic and symptomatic patients with MD.
- Using an increase in the integral area of absorbance as an indicator for identifying MD is controversial. During acute episodes of MD, WBT appears to be ineffective as a diagnostic tool.
- More research is needed to realize the potential of this technology in clinical applications.

## Author Contributions

XM conceptualized and drafted the manuscript. KZ, JY, and CH critically reviewed the literature and revised the draft manuscript. All authors contributed to the article and approved the submitted version.

## Funding

This study was funded by the Science and Technology Development Project of the Bureau of Science and Technology of Wuxi, China (Grant number CSZ0N1622).

## Conflict of Interest

The authors declare that the research was conducted in the absence of any commercial or financial relationships that could be construed as a potential conflict of interest.

## Publisher's Note

All claims expressed in this article are solely those of the authors and do not necessarily represent those of their affiliated organizations, or those of the publisher, the editors and the reviewers. Any product that may be evaluated in this article, or claim that may be made by its manufacturer, is not guaranteed or endorsed by the publisher.
